# A Cascade Approach to Valorizing *Camellia oleifera* Abel Shell: Ultrasound-Assisted Extraction Coupled with Resin Purification for High-Efficiency Production of Multifunctional Polyphenols

**DOI:** 10.3390/antiox14101192

**Published:** 2025-09-29

**Authors:** Jingyi Chen, Wei Li, Tao Liang, Yuting Yang, Rui Zhou, Rui Li, Daiyu Xie, Dayan Xiang, Shiling Feng, Tao Chen, Lijun Zhou, Chunbang Ding

**Affiliations:** School of Life Sciences, Sichuan Agricultural University, Ya’an 625000, China; camelia@stu.sicau.edu.cn (J.C.); liwei4@stu.sicau.edu.cn (W.L.); 2021316062@stu.sicau.edu.cn (T.L.); yangyuting1@stu.sicau.edu.cn (Y.Y.); 202204190@stu.sicau.edu.cn (R.Z.); lirui4@stu.sicau.edu.cn (R.L.); 2023316073@stu.sicau.edu.cn (D.X.); 2024216008@stu.sicau.edu.cn (D.X.); fsl@sicau.edu.cn (S.F.); chentao293@sicau.edu.cn (T.C.)

**Keywords:** *Camellia oleifera* Abel shell, polyphenols, ultrasound-assisted extraction, process optimization, bioactivity

## Abstract

*Camellia oleifera* Abel shell is an abundant lignocellulosic byproduct of the Chinese woody oil industry, which is currently underutilized. To achieve its high-value utilization, this study developed an innovative cascade process integrating ultrasound-assisted extraction and macroporous resin purification for the efficient preparation of purified polyphenols from the shell (P-CPCS). The major constituents were identified by quadrupole/Orbitrap high-resolution mass spectrometry (HPLC-Q-Exactive-MS: Biotech Pack Co., Ltd., Beijing, China) analysis. The optimized process significantly enhanced the polyphenol yield (40.05 ± 0.58 mg GAE/g dw) and purity (57.72%), surpassing conventional methods. P-CPCS exhibited exceptional multifunctional bioactivities, including potent antioxidant capacity (with low IC_50_ values against DPPH, ABTS^+^·, and ·OH radicals), effective tyrosinase inhibition (whitening effect), and significant bacteriostatic effects against various pathogens. Furthermore, P-CPCS notably suppressed the LPS-induced inflammatory response in RAW264.7 macrophages by reducing NO overproduction. This work highlights a novel and efficient strategy for upcycling agricultural waste into a high-performance natural antioxidant, positioning P-CPCS as a promising ingredient for applications in functional foods, cosmetics, and biomaterial stabilization.

## 1. Introduction

*Camellia oleifera* Abel., a woody oil plant of the Theaceae family native to China, is one of the world’s four major woody oil crops, along with olive, oil palm, and coconut [1]. The processing of its fruits yields a substantial amount of seed shells, accounting for approximately 60% of the fresh fruit weight [2]. Currently, these shells remain largely underutilized—commonly disposed of by incineration or limited to low-value lignin extraction—leading to significant resource waste and environmental concerns [3].

Plant polyphenols are recognized for their notable biological activities, including antioxidant, anti-inflammatory, and antimicrobial properties [4], presenting opportunities for innovative applications in sectors such as pharmaceuticals and cosmetics [5]. Previous studies have identified *C. oleifera* shells as a rich source of polyphenols, comprising 8.2–12.5% of their dry weight, and containing compounds such as rutin, hyperoside, licorice flavanone, proanthocyanidin, and isoquercitrin, with radical scavenging capacities reaching up to 89.3% [6].

The purification of bioactive compounds from crude plant extracts is crucial for both natural medicine research and industrial applications. However, conventional extraction techniques face considerable limitations: heat reflux may degrade thermolabile compounds due to prolonged high temperatures; supercritical CO_2_ extraction entails high operational costs [7]; and enzyme-assisted extraction suffers from issues related to stability and scalability [8]. Moreover, most existing studies focus merely on crude extract preparation, which falls short of industrial requirements for high-purity polyphenol products. These attributes make the UAE a highly promising method for industrial-scale polyphenol extraction.

In this context, ultrasound-assisted extraction (UAE) offers distinct advantages, such as reduced solvent consumption (by 30–50%), shorter processing times (over 60% faster), lower energy input, and improved preservation of bioactive compounds—aligning effectively with green chemistry principles [9,10].

This study aims to optimize UAE for the recovery of polyphenols from *C. oleifera* shells using Box–Behnken response surface methodology (RSM), examining the interactive effects of ultrasonic time, ethanol concentration, and liquid-to-material ratio on extraction yield. The obtained polyphenols were further purified through dynamic adsorption with macroporous resin and characterized via high-performance liquid chromatography coupled with quadrupole/Orbitrap high-resolution mass spectrometry (HPLC-Q-Exactive-MS). In vitro assays were conducted to assess their antioxidant, anti-inflammatory, whitening, and antimicrobial properties. Our work not only establishes an efficient and scalable UAE protocol for the valorization of *C. oleifera* shells but also provides a methodological framework for the high-value, sustainable utilization of analogous agroforestry byproducts. This approach underscores their significant potential in enhancing antioxidant performance, thereby supporting the application of natural antioxidants in the realm of sustainable resource recovery.

## 2. Materials and Methods

### 2.1. Chemicals and Reagents

All chemicals and reagents used were of analytical grade unless otherwise stated. ABTS^+^·, DPPH, sodium selenite (chemically pure), trichloroacetic acid, trichloromethane, anhydrous ethanol, n-butanol, nitric acid, sodium carbonate, anhydrous dextrose, phenol, sulfate, cholesterol, magnesium sulfate, calcium chloride, methanol, and acetonitrile were sourced from Chengdu Cologne Chemical Co., Ltd. (Chengdu, China). The CCK-8, ROS, and Griess detection kits were procured from Chengdu Gaoxin District Yiyuan Experimental Consumables Business Department (Chengdu, China). The RPMI-1640 medium was purchased from HyClone (a Cytiva company) (Marlborough, MA, USA). Tryptone, peptone, yeast extract, and agar powder were obtained from Oxoid Ltd. (Hampshire, IL, USA). The HPLC-Q-Exactive-MS analysis was performed on an instrument from Biotech Pack Co., Ltd. (Beijing, China).

Microbial strains (*Escherichia coli*, *Staphylococcus aureus*, *Pseudomonas aeruginosa*, *Bacillus subtilis*, and *Candida albicans*) were obtained from the Department of Bioengineering and Applied Biology, Sichuan Agricultural University. Human umbilical vein endothelial cells (HUVECs) and mouse macrophage cells (RAW264.7) were obtained from the Chinese Academy of Sciences (Shanghai, China).

### 2.2. Sample Preparation

Fresh *C. oleifera* shells were collected from the experimental field in Tianquan County, Sichuan Province, in 2023. The samples were washed, dried, ground (60-mesh sieve), and stored at −20 °C until use.

### 2.3. UAE of Polyphenols

Polyphenol extraction was performed using an ultrasonic cleaner (KH7200DB, Kunshan Wo Chuang Ultrasonic Instruments Co., Ltd., Kunshan, China) with fixed parameters: power (270 W) and temperature (55 °C). To investigate the effects of different factor levels on the polyphenol extraction rate (Table 1).

#### 2.3.1. Box–Behnken Design (BBD)

A Box–Behnken design (three factors, three levels) was then employed for optimization (Table 2).

#### 2.3.2. Polyphenol Quantification

Total polyphenol content (TPC) was determined using the Folin–Ciocalteu method [11], according to Equation (1):

Yield (%) = (C × V × N)/(M × 1000)(1)

where C = concentration (μg/mL), V = volume (mL), N = dilution factor, and M = sample weight (g).

### 2.4. Optimization of Polyphenol Purification Process

#### 2.4.1. Preparation of Crude Polyphenol

The extract was concentrated by rotary evaporation and freeze-dried.

#### 2.4.2. Static Adsorption–Desorption Experiments of Macroporous Resins

##### Determination of Polyphenol Adsorption and Desorption Rates

Weighed 1.0 g (dry weight) of the pretreated macroporous resin was mixed with 25 mL of the *C. oleifera* polyphenol solution to be purified (9 mg/mL) in a sealed container, then shaken at 25 °C at 150 rpm for 24 h. The polyphenol content of the supernatant was determined. After filtration, the resin was washed with ultrapure water to remove surface moisture and then mixed with 25 mL of 70% ethanol. The mixture was sealed and shaken under the same conditions for 24 h before determining the polyphenol content in the supernatant. The adsorption rate was calculated according to Equation (2), and the desorption rate according to Equation (3).

A (%) = (C_0_ − C_1_)/C_0_ × 100 (2)

D (%) = C_2_/(C_0_ − C_1_) × 100 (3)

where A = adsorption rate; D = desorption rate; C_0_ = content before adsorption (mg/mL); C_1_ = content after adsorption (mg/mL); C_2_ = content after desorption (mg/mL).

##### Construction of Static Adsorption–Desorption Profiles

Adsorption was performed by mixing 1.0 g of resin with 25 mL of a 9 mg/mL (pH 2) solution of polyphenol to be purified under the same conditions. The polyphenol concentration in the supernatant was measured every 30 min until equilibrium was reached. The adsorption rate was calculated, and the adsorption kinetic curve was plotted. After adsorption, the resin was washed with ultrapure water, and desorption was performed by adding 25 mL of 70% ethanol under the same conditions. The polyphenol concentration was measured at 20 min intervals until equilibrium was reached. The desorption rate was calculated, and the desorption kinetic curve was plotted.

##### Study on the Adsorption and Desorption Characteristics of Macroporous Resin for Polyphenols

Weighing 1.0 g of pretreated resin and 25 mL of a solution of polyphenol to be purified with different concentrations (3–18 mg/mL, pH 2) or different pH (2–8, 9 mg/mL), the adsorption was carried out at 25 °C and 150 rpm. The adsorption rate was determined to evaluate the effect of concentration and pH of the upper sample solution. The adsorption saturated resin was washed with ultrapure water and desorbed with 25 mL of ethanol solutions at varying concentrations (10–90%) under the same conditions for 24 h. The desorption rate was calculated by determining the polyphenol content of the desorbed liquid to determine the optimal desorption conditions.

#### 2.4.3. Dynamic Adsorption–Desorption Experiments of Macroporous Resin

A certain amount of pretreated macroporous resin was loaded into a 1.5 cm × 30 cm chromatography column according to the wet loading method and pressed with ultrapure water until the height of the column bed remained unchanged (the column volume was 15 mL).

##### Determination of Leakage Curve

400 mL of the liquid to be purified was up-sampled at a flow rate of 1 mL/min, the effluent was picked up, and another centrifuge tube was changed after every 7.5 mL (0.5 BV) until the polyphenol concentration of the effluent reached 10% of the concentration of the up-sampled liquid (the leakage point), and the leakage curve was plotted.

##### Determination of Elution Curve

Take 100 mL of *C. oleifera* shell polyphenol to be purified solution at a rate of 1 mg/mL for the upper sample after the ultrapure water cleaning until the effluent is colorless. Elution was carried out with 70% ethanol at 0.5, 1, 1.5, and 2 mL/min, respectively. The polyphenol content of the eluate was determined, and the elution curve was plotted.

##### Effect of Elution Speed on the Desorption Efficiency of Macroporous Resin

The *C. oleifera* shell polyphenol was purified in a solution at a speed of 1.0 mL/min for the up-sampling, and the deionized water was cleaned until the effluent became colorless. Using 70% ethanol as the eluent, the elution was carried out at a speed of 1.0 mL/min, and the polyphenol concentration of the eluent was measured to determine the elution speed.

##### Validation of the Optimal Process

According to the optimal process conditions derived from the screening, three sets of parallel tests were conducted to determine the polyphenol content of the eluate and calculate the purity and recovery of CPCS before and after purification.

Purity (%) = total polyphenol mass of eluate/total solids mass × 100(4)

Recovery (%) = total polyphenols in eluent/total polyphenol mass in upper sample × 100(5)

### 2.5. HPLC-Q-Exactive-MS Analysis

The analysis of CPCS was performed using HPLC-Q-Exactive-MS. The separation was carried out on a Waters HSS T3 column (2.1 mm × 100 mm, 1.7 μm) with a column temperature of 40 °C. The flow rate was set at 0.3 mL/min, and the injection volume was 5 μL. Mobile phase A consisted of an aqueous formic acid solution (0.1%), while mobile phase B was pure methanol (100%). The elution program was as follows: 0–1 min, 2–2% B; 1–5.5 min, 2–100% B; 5.5–14 min, 100–100% B; 14–15 min, 100–2% B; 15–16 min, 2–2% B.

### 2.6. In Vitro Antioxidant Capacities

#### 2.6.1. ABTS^+^·/DPPH Radical Scavenging Capacity

ABTS^+^· radical scavenging capacity of the extracts was determined according to a study provided by Re et al. [12] and optimized. ABTS^+^· stock solution (7.4 mmol/L) and potassium persulfate stock solution (2.6 mmol/L) were mixed in equal quantities and left in the dark for 12 h. Before use, the ABTS^+^· radical solution was diluted with PBS buffer to an absorbance of 0.70 ± 0.02 at 734 nm. 100 μL solution was added to 96 wells with an equal volume of extraction sample solution (or Vc). The absorbance of the mixture was measured at 734 nm after storage in the dark for 6 min. The clearance was calculated according to Equation (6):

ABTS^+^· radical scavenging activity (%) = [1 − (A_1_ − A_2_)/A_0_] × 100% (6)

where A_0_ = absorbance of blank group; A_1_ = absorbance after reaction with ABTS^+^· working solution; A_2_ = background absorbance after mixing with ultrapure water.

DPPH radical scavenging assay of the extract was detected with a slight adjustment to the method of Ou et al. [13]. The polyphenol extracts or Vc (70 μL) and 0.4 mmol/L DPPH solution (140 μL) were added into a 96-well plate and shaken and mixed, then left in the dark for 30 min. The absorbance of the mixture was measured at 517 nm. The clearance rate was calculated according to the equation (same as the ABTS^+^· equation).

#### 2.6.2. Hydroxyl Radical (·OH) Scavenging

An adapted experiment to assess the ability to scavenge ·OH radicals was carried out by the procedure outlined by Wang et al. [14] and optimized: 0.50 mL of 0.15 mol/L FeSO_4_ was mixed with 0.50 mL of 9 mmol/L H_2_O_2_ solution, and incubated in the lucifuge for 5 min, then 0.20 mL of 2 mmol/L salicylic acid-ethanol solution and 0.10 mL of the extracted sample solution or Vc was added in turn, following thorough mixing and incubating in the lucifuge at 37 °C for 60 min. Determine the absorbance of the solution at a wavelength of 510 nm. The scavenging activity was calculated according to Equation (7):

Rate of clearance (%) = [1 − (A_1_ − A_2_)/A_0_] × 100 (7)

where A_0_ = absorbance of blank group; A_1_ = absorbance of sample; A_2_ = background absorbance of sample.

#### 2.6.3. Reducing Power

A volume of 0.2 mL of sample or VC solution was mixed with 0.5 mL of 0.2 mol/L PBS (pH 7.4) and 0.5 mL of 1% potassium ferricyanide solution. After this mixture was heated in a water bath at 50 °C for 20 min, a volume of 0.5 mL of 10% TCA was added and cooled at 0 °C for 5 min. 0.4 mL of 0.1% ferric chloride solution and 2 mL of distilled water were added, and then the solution absorbance was measured spectrophotometrically at 700 nm.

#### 2.6.4. Cytoprotection Against Oxidative Stress

##### HUVEC Culture and Sample Preparation

HUVEC were cultured in RPMI-1640 medium containing 10% fetal bovine serum and 1% penicillin (37 °C, 5% CO_2_). When the cell density reached 85%, the cells were digested with trypsin for 2 min, and fresh medium was used to terminate the digestion and collect the cells, which were washed twice with PBS, centrifuged at 1200 r/min for 3 min, and passaged at a 1:2 ratio.

The experimental samples were dissolved in DMSO and prepared as a 1600 μg/mL master mix (final concentration of DMSO < 0.05%) using RPMI-1640 medium, filtered through a 0.45 μm filter membrane, and then diluted twofold to 25 μg/mL for reserve.

##### Cytotoxicity Assay (CCK-8 Method)

The CCK-8 assay was employed to assess the effect of purified polyphenols from *C. oleifera* shells on the viability of HUVEC (using RPMI-1640 medium containing 10% fetal bovine serum and 1% penicillin, routinely cultured at 37 °C in a 5% CO_2_ incubator) were inoculated in 96-well plates at a density of 5 × 10^3^ cells/well, and cultured for 12 h to affix the walls, different concentrations of polyphenols were added and treated for 24 h. The medium was aspirated and discarded, and 100 μL of fresh medium containing 10% CCK-8 was added. After incubation for 45 min, the absorbance at 450 nm was measured. The cell viability was calculated by the following Equation (8):

The ratio of inhibition (%) = (OD_i_ − OD_0_)/(OD_j_ − OD_0_) × 100 (8)

##### H_2_O_2_ Induced Damage

After pre-incubation of HUVEC with polyphenols for 24 h, 100 μM of H_2_O_2_ solution was added to 100 μL of culture for 5 h. Cell viability was determined by Equation (8).

##### Measurements of Intracellular Reactive Oxygen Species (ROS)

Referring to the method of J. Das et al. [15]. The HUVEC were exposed to DCFH-DA, and the fluorescence intensity was subsequently analyzed by flow cytometry. The overall intracellular ROS levels were quantified as the mean DCF fluorescence intensity of the cells.

##### Detection of Apoptosis

Referring to the method of Yean Leng Loke et al. [16]. HUVEC were seeded in 96-well plates at 50 μL/well at a density of 1 × 10^5^ cells/mL and placed in a 37 °C, 5% CO_2_ incubator for 12 h to make the cells adhere. 50 μL of different concentrations of the sample solution to be tested was added to each well to continue incubation for 24 h. Then, the medium was discarded, and 100 μL of 100 μM H_2_O_2_ solution was added to each well to induce oxidative damage, and the incubation was continued for 5 h. The cells were collected with 1× binding solution.

After the cells were collected, they were resuspended to a volume of 100 μL with 1× binding buffer, and 5 μL of Annexin V-FITC (Annexin V Conjugates for Apoptosis Detection, Invitrogen^TM^, Waltham, MA, USA) was added and incubated for 10 min at room temperature away from light, followed by the addition of 1 μL of propidium iodide (PI, 100 μg) and 1 μL of propylene iodide (PI, 100 μg). Finally, 300 μL of binding buffer was added to dilute the samples, which were immediately analyzed using a flow cytometer (Accuri C6).

### 2.7. Tyrosinase Inhibitory Activity

Based on the tyrosinase dopa rate oxidation method [17], the reaction group was constructed by combining 1 mg/mL L-dopa solution (pH 6.8 PBS preparation), 100 U/mL tyrosinase solution (stored on ice), and 1 mg/mL sample solution according to the system in Table 3, and the absorbance was measured at 475 nm after incubation at 37 °C and protected from light for 10 min to calculate the rate of inhibition according to Equation (9):

Ainhibition rate (%) = (A_1_ − A_2_) − (A_2_ − A_3_)]/(A_0_ − A_1_) × 100 (9)

### 2.8. Determination of Bacteriostatic Activity

#### 2.8.1. Zone of Inhibition Measurement

This study examined the antimicrobial effects of the sample solutions against bacterial strains, namely Gram-positive *S. aureus* ATCC 25923 and Gram-negative *E. coli* ATCC 25922, which were determined by the agar diffusion method [18], and all the experiments were repeated three times.

#### 2.8.2. MIC Determination

The minimum inhibitory concentration (MIC) of the optimized sample solution was calculated using the two-fold dilution method (diluting the sample solution to a final concentration range of 16–0.125 mg/mL).

### 2.9. Anti-Inflammatory Activity

#### 2.9.1. RAW264.7 Cell Culture and Sample Preparation

The RAW264.7 cell culture system is the same as Section 2.6.4 HUVEC Culture and Sample Preparation, and passaging was performed when the cell density reached 70%.

Samples were prepared as in Section 2.6.4 HUVEC Culture and Sample Preparation (final dilution concentration of 50 μg/mL).

Dexamethasone (50 μg/mL, dissolved in anhydrous ethanol, final concentration < 0.05%) was used as a positive control.

#### 2.9.2. Cell Viability Assay

Determination of RAW264.7 cell viability using the method described in Section 2.6.4 Cytotoxicity Assay (CCK-8 Method).

#### 2.9.3. Determination of NO Content (Griess Method)

1 × 10^5^ cells/mL of RAW264.7 cells were seeded into 96-well plates (50 μL/well), pre-cultivated in an incubator with 5% CO_2_ at 37 °C for 12 h, and then added with different concentrations of sample solution (50 μL/well). Sodium nitrite was used as the standard, and the standard curve was plotted according to the instructions of the reagent kit, and NO content was calculated according to Equation (10):

NO content (μm) = (A_1_ − A_0_)/K (10)

where A_1_ = absorbance after reaction with Griess reagent; A_0_ = background absorbance of the medium with Griess reagent; K = slope of the standard curve fitted with sodium nitrite.

### 2.10. Statistical Analysis

The data were collected in triplicate and presented as mean ± standard deviation (SD). Statistical significance (*p* < 0.05) was identified by one-way analysis of variance (ANOVA) using GraphPad Prism9 (GraphPad Software, Inc., La Jolla, CA, USA).

## 3. Results

### 3.1. Optimization of C. oleifera Shell Polyphenol Component (CPCS) Extraction

#### 3.1.1. Single-Factor Analysis

The pre-experiments revealed that the TPC values increased with increasing ultrasound power from 100 W to 270 W and then decreased when the ultrasound power exceeded 270 W. Preliminary experiments indicated that the yield of CPCS increased with rising ultrasonic temperature, peaking at 50 °C. The effects of ethanol concentration, ultrasonic time, and L/M ratio on the extraction rate of CPCS were systematically investigated.

##### Ethanol Concentration

**Figure 1:**
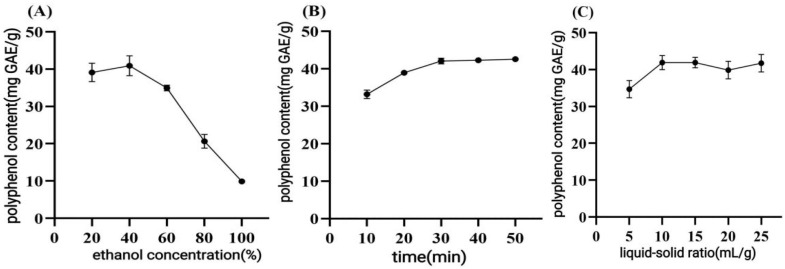
(**A**) Ethanol concentration, (**B**) extraction time, (**C**) effect of L/M ratio on CPCS extraction rate.

As shown in Figure 1A, the extraction yield increased significantly with ethanol concentration in the range of 0–40%, reaching a maximum value of 38.92 mg GAE/g at 40% ethanol. When the ethanol concentration exceeded 40%, the extraction efficiency declined.

##### Ultrasound Time

Figure 1B illustrates that the extraction yield increased with ultrasound time up to 30 min (39.15 mg GAE/g) and plateaued thereafter.

##### L/M Ratio

The L/M ratio significantly influenced extraction efficiency (Figure 1C). The optimal yield (38.76 mg GAE/g) was achieved at 10 mL/g.

#### 3.1.2. BBD Optimization and ANOVA Results

Based on the Box–Behnken experimental (Table 4) design and single-factor analysis results, three key factors affecting polyphenol extraction yield were identified: ultrasonic time (A), ethanol concentration (B), and L/M ratio (C). The second-order polynomial model was established as follows:

Y = 38.96 + 0.66A − 2.68B + 3.44C + 0.34AB + 0.20AC + 0.26BC − 0.13A^2^ − 3.74B^2^ − 3.26C^2^ (11)

where Y = content, A = ultrasound time, B = ethanol concentration, C = L/M ratio.

Table 5 presents the ANOVA results. The model showed an F-value of 108.58 with *p* < 0.0001, indicating highly significant model adequacy (*p* < 0.01). The lack-of-fit value of 0.3096 (*p* > 0.05) demonstrated it was not statistically significant compared to pure error. The determination coefficient (R^2^ = 0.9929) and adjusted R^2^ (0.9837) indicated that the model explained 98.37% of the response variability, confirming a good fit. The low coefficient of variation (CV = 1.18%, <5.0%) confirmed model reproducibility. The significance analysis of the experimental factors demonstrated that linear terms (A: time, B: ethanol concentration, C: solid–liquid ratio) and quadratic terms (B^2^, C^2^) had extremely significant effects on the CPCS extraction yield (*p* < 0.01). In contrast, the AC interaction and A^2^ quadratic term showed a considerable influence (*p* < 0.05). Other interaction terms (AB, BC) were statistically insignificant (*p* > 0.05), collectively indicating a complex nonlinear relationship between process parameters and extraction efficiency. Based on the comparative F-values within the experimental design space, the relative importance of the factors followed the order: L/M ratio > extraction time > ethanol concentration, providing critical guidance for process optimization.

#### 3.1.3. RSM Analysis

**Figure 2:**
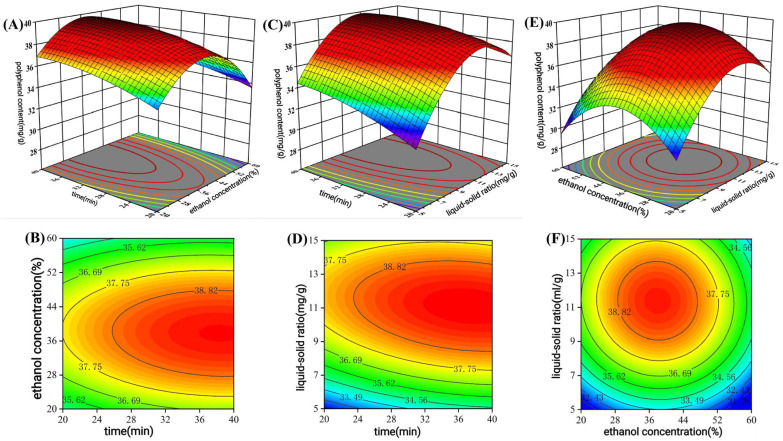
Three-dimensional plots and contour plots of response surface results. Three-dimensional plots (**A**) and contour plots (**B**) for extraction time and ethanol concentration interaction; 3D plots (**C**) and contour plots (**D**) for extraction time and L/M ratio concentration interaction; 3D plots (**E**) and contour plots (**F**) for extraction time and L/M ratio interaction.

Figure 2 presents the response surface plots and contour maps obtained using Design Expert 10 software based on the binary polynomial regression equation. The steeper the slope of the response surface and the closer the contour map is to an ellipse, the more significant the interaction between the two factors, indicating a greater impact on the extraction yield of purified polyphenols from *C. oleifera* shell (P-CPCS). The downward-opening directions of all three response surface plots suggest that the response values reach their maximum within the ranges of the three factors. In the three-dimensional response surface plots (A, C, E), a color gradient transitioning from blue to red corresponds to an increase in the predicted polyphenol yield (mg GAE/g dw). The corresponding contour plots (B, D, F) use elliptical or circular shapes to represent the interaction strength between factors, where elliptical contours indicate a significant interaction. From Figure 2A, it can be observed that the extraction yield of CPCS increases with the increase in time (A) and ethanol concentration (B). When the ethanol concentration exceeds 40%, the extraction yield of P-CPCS decreases. Figure 2C shows that the interaction between time (A) and L/M ratio (C) has a significant effect on the extraction yield of P-CPCS. The corresponding response surface exhibits the steepest slope, and the contour lines form an ellipse (Figure 2D), indicating the strongest interaction. The extraction rate of P-CPCS increases with the increase in time and L/M ratio, and slightly decreases with the increase in L/M ratio to a certain level. The steeper slope of the L/M ratio variation surface compared to that of the time variation surface suggests that the L/M ratio in the interaction has a greater influence on the extraction yield of polyphenols than time does, which is also consistent with the results in the ANOVA table. In Figure 2E, as ethanol concentration (B) and L/M ratio (C) increase, the polyphenol extraction yield initially rises and then declines slightly. The contour lines form a circular shape (Figure 2F), indicating a weak interaction between these two factors.

After the response surface optimization of the extraction conditions of the TPC of *C. oleifera* shells, the optimal extraction process conditions were determined as follows: extraction time of 40 min, ethanol concentration of 37.58%, L/M ratio of 1:11.01, and the extraction rate of polyphenols reached 39.88 mg GAE/g dw. Based on practical considerations, the conditions were adjusted to an extraction time of 40 min, ethanol concentration of 38% and L/M ratio of 1:11; the actual extraction rate of total phenols was 40.05 ± 0.58 mg GAE/g dw, showing no significant difference. This indicates that the extraction process optimized by the response surface model is stable and reliable.

### 3.2. Purification of CPCS

#### 3.2.1. Analysis of Static Adsorption–Desorption Test Results of Macroporous Resin

##### Static Adsorption and Desorption Curves of AB-8 Macroporous Resin

**Figure 3:**
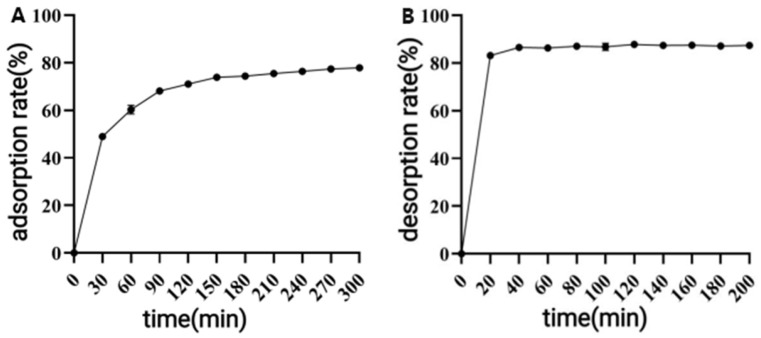
Static adsorption curve (**A**) and static desorption curve (**B**) of AB-8 macroporous resin.

As shown in Figure 3, the adsorption rate of polyphenols by the AB-8 macroporous resin increased rapidly within 0–90 min, followed by a slower progression until reaching adsorption equilibrium at 150 min with an adsorption ratio of 73.85%. Desorption achieved 82.43% efficiency within just 20 min and essentially reached equilibrium (86.05%) by 40 min. These results demonstrate that the AB-8 macroporous resin exhibits efficient adsorption–desorption performance for CPCS, indicating its suitability for CPCS separation and purification.

##### Effect of Sample Concentration

**Figure 4:**
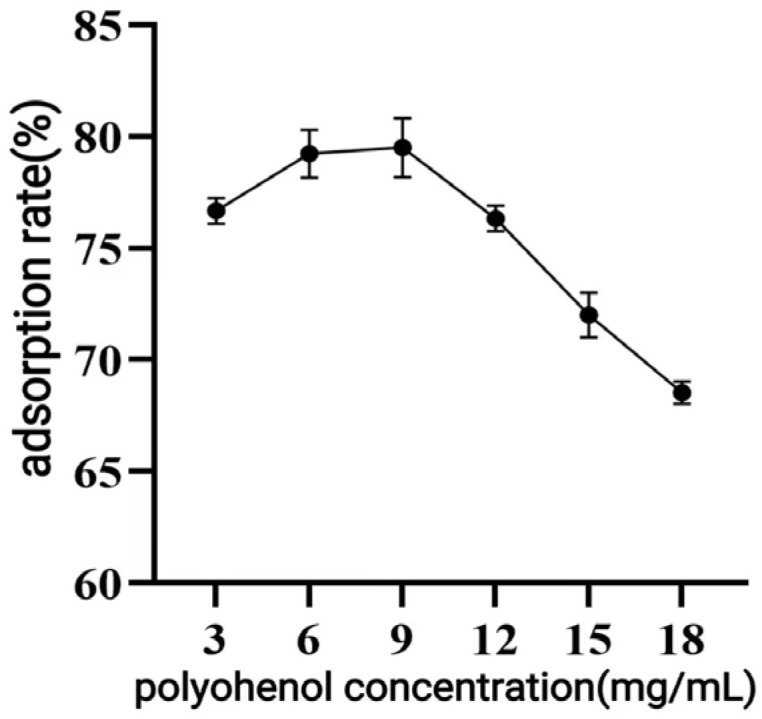
Effect of loading concentration on the adsorption effect of macroporous resin.

As shown in Figure 4, the adsorption rate of *C. oleifera* shell polyphenols gradually increased as the sample concentration rose from 3 to 9 mg/mL, peaking at 9 mg/mL. Therefore, 9 mg/mL was identified as the optimal sample concentration.

##### Effect of pH

**Figure 5:**
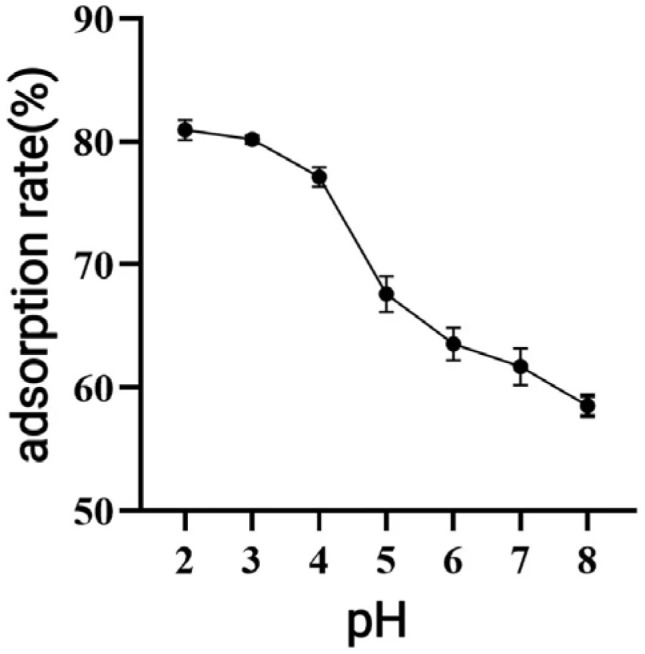
Effect of sample pH on the adsorption effect of macroporous resin.

As demonstrated in Figure 5, the adsorption rate decreased as pH increased, with the highest adsorption observed under acidic conditions at pH = 2.

##### Effect of Ethanol Concentration

**Figure 6:**
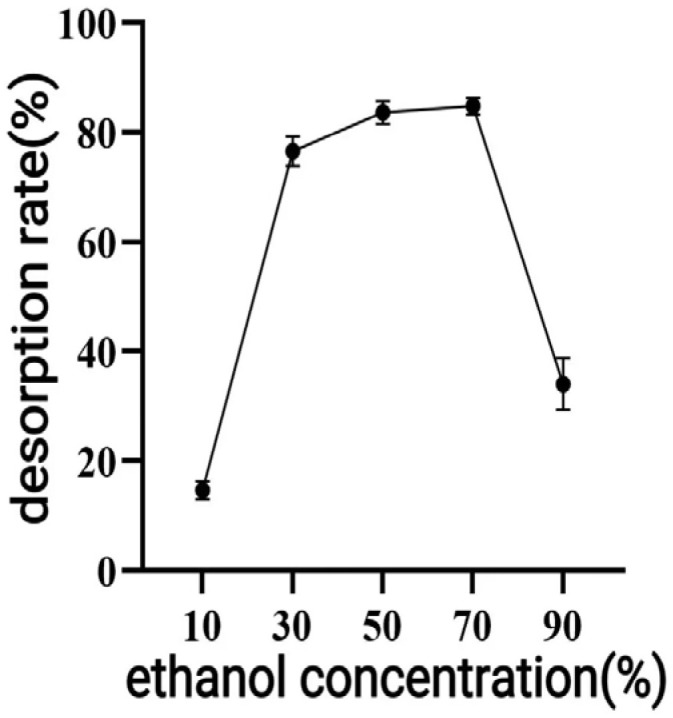
Effect of ethanol concentration on the adsorption effect of macroporous resin.

As shown in Figure 6, the desorption rate increased with ethanol concentration until it reached a maximum at 70%. However, the yield started to drop as the ethanol concentration rose above 70%. Consequently, 70% ethanol was selected as the optimal eluent.

#### 3.2.2. Analysis of Dynamic Adsorption–Desorption Test Results of Macroporous Resin

##### Investigation of Leakage Curve

**Figure 7:**
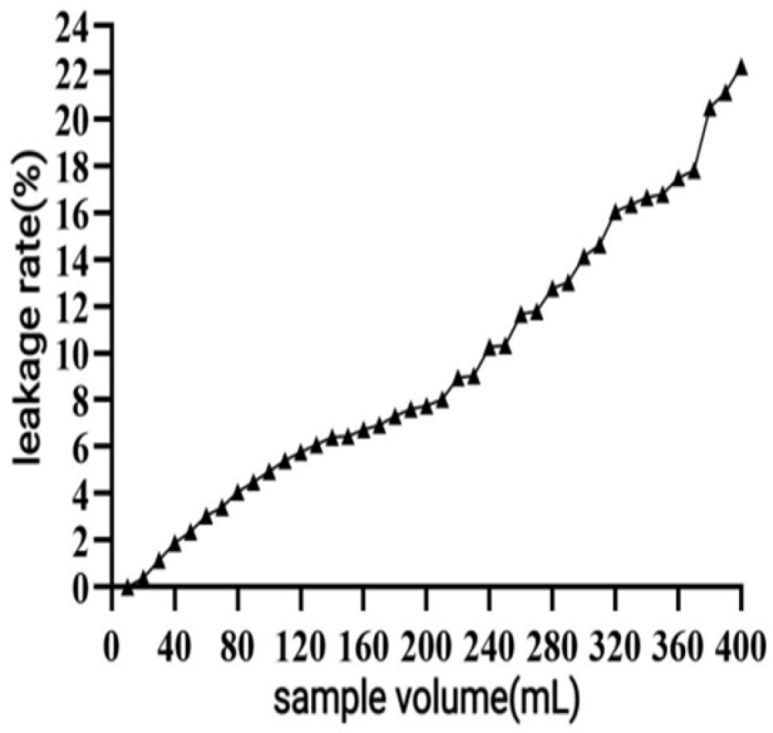
Leakage curve of AB-8 large hole resin.

The leakage curves for the macroporous resin are shown in Figure 7. The leakage point was reached when the up-sampling volume was increased to 220 mL, and the leakage was 10.28%. Subsequently, the leakage rate increased significantly. Therefore, the maximum sample volume was determined to be 220 mL (Figure 7).

##### Effect of Elution Speed

**Figure 8:**
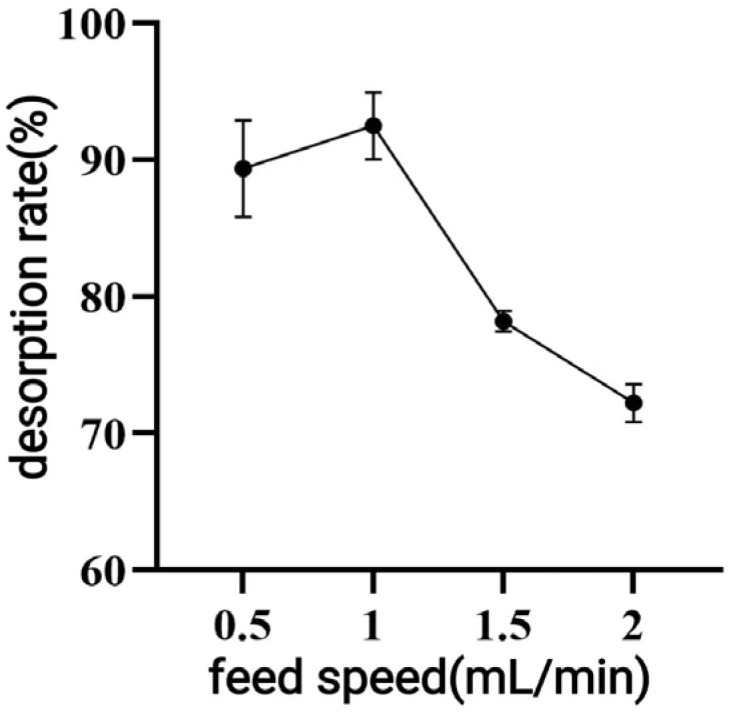
Effect of elution velocity on the desorption effect of macroporous resin.

The elution rate significantly affected resin adsorption, and good desorption was achieved at both 0.5 and 1 mL/min. However, when the flow rate exceeded 1.5 mL/min, the desorption rate decreased significantly (*p* < 0.05). Therefore, 1 mL/min was chosen as the optimal elution rate (Figure 8).

##### Elution Curve

**Figure 9:**
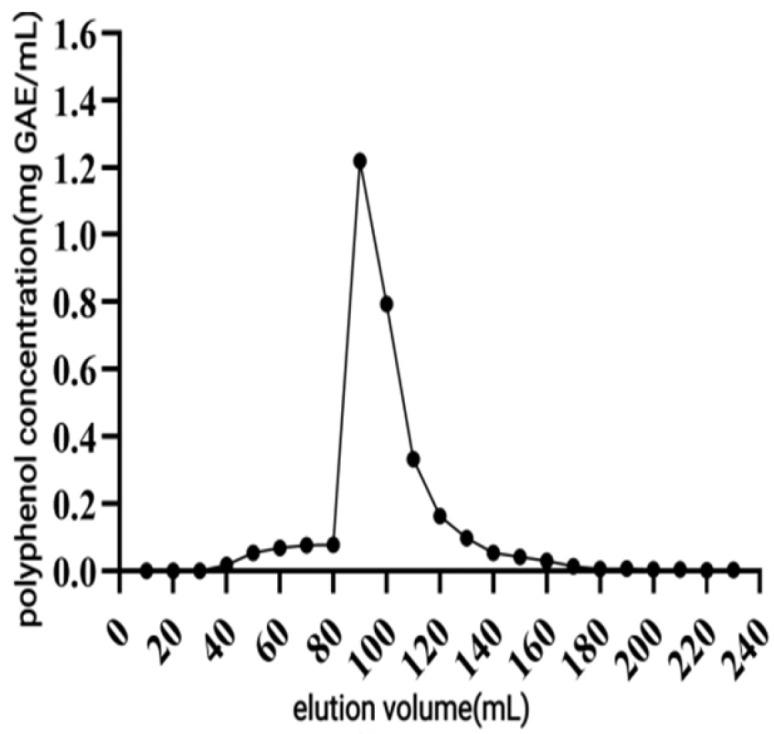
Elution curve of macroporous resin.

When the elution volume reached 90 mL, the polyphenol concentration reached the maximum value of 1.22 mg GAE/mL, then the polyphenol concentration gradually decreased. At 160 mL, over 90% of the polyphenols had been eluted. Further increasing the volume to 180 mL resulted in the polyphenol concentration approaching 0, indicating that polyphenols had been basically eluted out. Considering the time and cost, 160 mL was chosen as the elution volume (Figure 9).

#### 3.2.3. Purification Process Validation

Under the optimized conditions, the polyphenol purity in CPCS increased from 24.93% to 57.72%, while the recovery rate reached 77.19%. These findings confirm that AB-8 macroporous resin has a significant purification effect on CPCS.

### 3.3. CPCS Component Analysis

**Figure 10:**
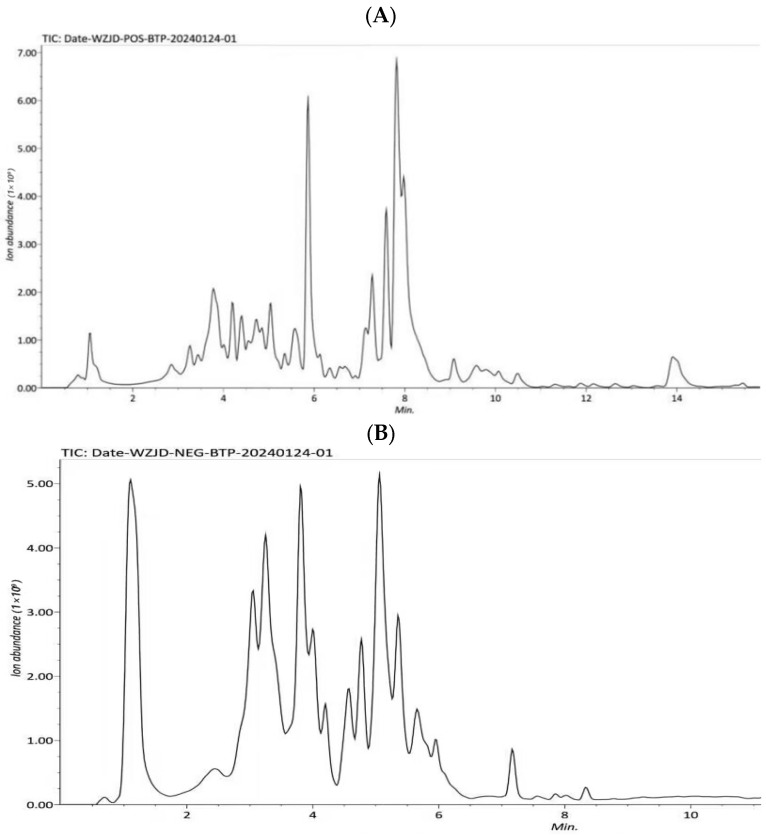
The total ion currents for purified polyphenols from *C. oleifera* shell (P-CPCS): (**A**) positive ion mode; (**B**) negative ion mode.

HPLC-Q-Exactive-MS analysis in both positive and negative ion modes successfully identified a total of 20 polyphenolic compounds in P-CPCS (Figure 10, Table 6), primarily categorized into flavonoids (15 compounds) and phenolic acids (5 compounds).

### 3.4. Biological Activities of CPCS

#### 3.4.1. Antioxidant Capacity

**Figure 11:**
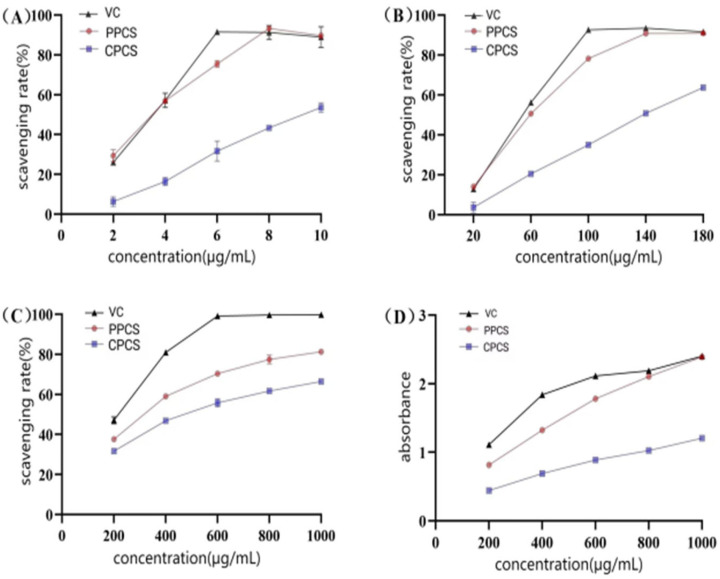
Antioxidant activity of polyphenols from shell of *C. oleifera* before and after purification: (**A**) ABTS^+^· radical scavenging activity; (**B**) DPPH radical scavenging activity; (**C**) OH radical scavenging activity; (**D**) total reducing force.

Vc was used as a positive control to investigate the DPPH·, ABTS^+^·, and ·OH before and after purification of polyphenols. Scavenging activities and total reducing power were studied. The results demonstrated that both the polyphenols before and after purification exhibited strong free radical scavenging capacity and high reducing power in a dose-dependent relationship. And the antioxidant activity of P-CPCS was significantly enhanced after purification. As shown in Figure 11, the IC_50_ values of P-CPCS against DPPH·, ABTS^+^· were 55.59 μg/mL, 3.26 μg/mL, respectively, which were significantly better than those of the unpurified extracts (135.6 μg/mL, 9.23 μg/mL) and close to Vc. The DPPH· scavenging ability of P-CPCS at a concentration of 140 μg/mL, and ABTS^+^· scavenging ability was close to that of Vc after 8 μg/mL. The results showed that P-CPCS had good antioxidant activity, with purified fractions demonstrating significantly enhanced efficacy.

#### 3.4.2. Cellular Protection Against Oxidative Damage

**Figure 12:**
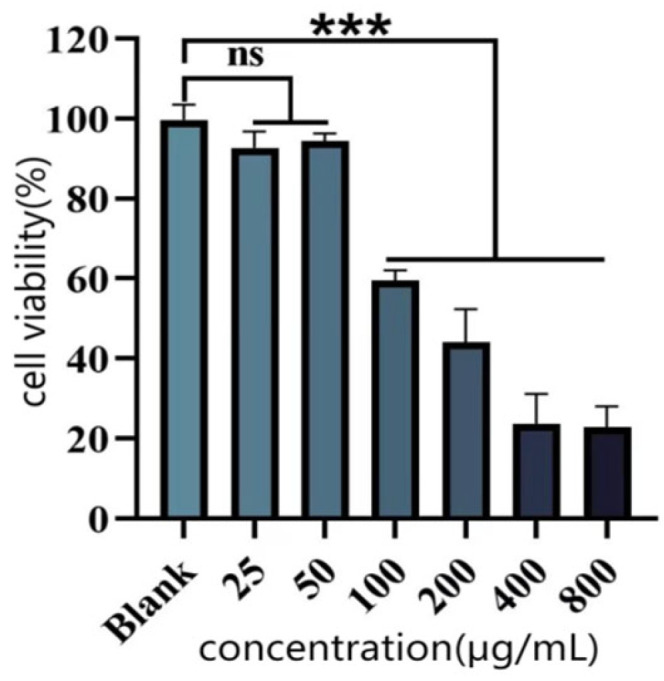
Effect of purified polyphenols from *C. oleifera* shell (P-CPCS) on the viability of HUVEC. Note: *** *p* < 0.001, ns, not significant.

As shown in Figure 12, HUVEC cell viability showed no significant difference from the blank control group when treated with 25–50 μg/mL P-CPCS. After 100 μg/mL, cell viability decreased significantly. Therefore, 25 μg/mL and 50 μg/mL were chosen for subsequent experiments.

**Figure 13:**
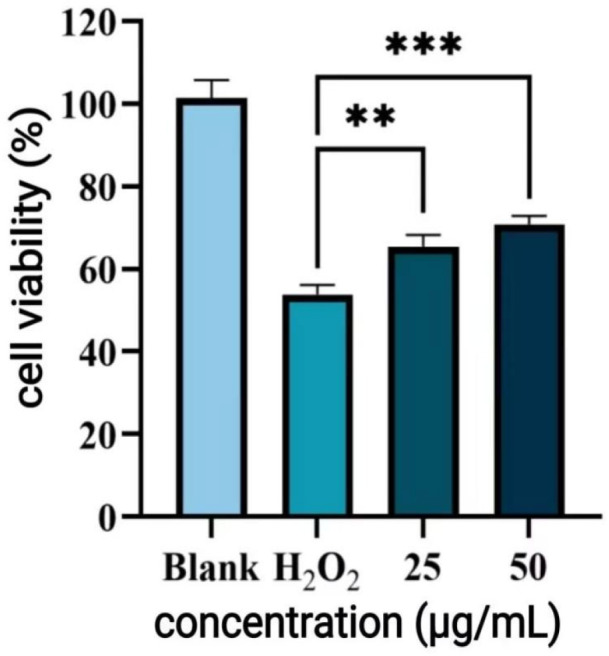
Effect of purified polyphenols from *C. oleifera* shell (P-CPCS) on the viability of HUVEC under oxidative stress. Note: *** *p* < 0.001, ** *p* < 0.01.

H_2_O_2_ is a strong oxidant widely used to induce oxidative stress in cells and tissues. As shown in Figure 13, treatment with 100 μM H_2_O_2_ reduced cell viability to 54.62%, indicating severe oxidative damage. Treatments with 25 μg/mL and 50 μg/mL P-CPCS, cell viability was significantly increased to 63.77% and 70.51%. This indicates that P-CPCS exhibit protective effects against oxidative cellular damage.

**Figure 14:**
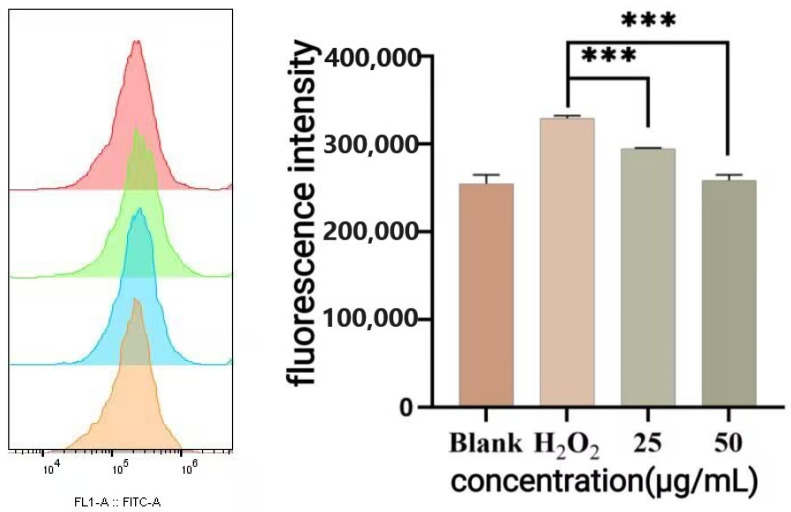
Changes in the intracellular ROS levels. Note: *** *p* < 0.001. The shift in fluorescence intensity (x-axis) reflects the amount of intracellular ROS, while the color gradient from blue to red indicates increasing cell density.

As shown in Figure 14, the H_2_O_2_-treated group exhibited a significant increase in ROS levels compared to the blank group. Compared with the oxidative damage model group, 25 μg/mL and 50 μg/mL P-CPCS treatment significantly reduced the ROS accumulation. These results demonstrate that P-CPCS could effectively reduce the ROS level of cells under oxidative stress, thus alleviating oxidative damage.

**Figure 15:**
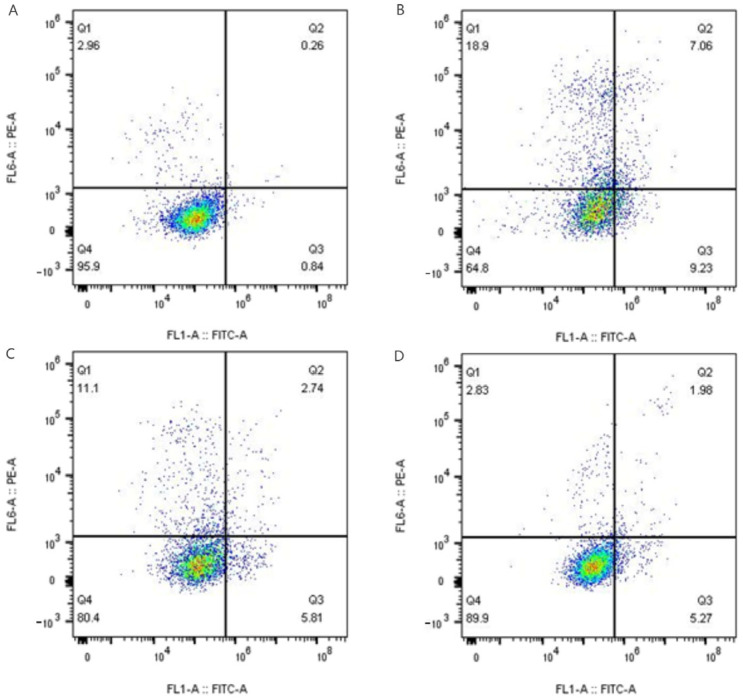
Annexin V-FITC/PI double staining flow cytometry showing HUVEC apoptosis in (**A**) blank control, (**B**) H_2_O_2_-induced model group, (**C**) 25 μg/mL, and (**D**) 50 μg/mL P-CPCS treatment groups. The quadrants delineate cell populations as follows: Q1: necrotic; Q2: late apoptotic; Q3: early apoptotic; Q4: viable. The accompanying percentages quantify the distribution of cells among these states.

Annexin V-FITC/PI double staining flow analysis (Figure 15) showed that H_2_O_2_ treatment increased the apoptosis rate to 14.68%, whereas 25 μg/mL and 50 μg/mL P-CPCS treatments, respectively, reduced the apoptosis rate to 8.28% and 7.33%, suggesting that P-CPCS effectively inhibited oxidative stress-induced apoptosis.

#### 3.4.3. Anti-Inflammatory Activity

**Figure 16:**
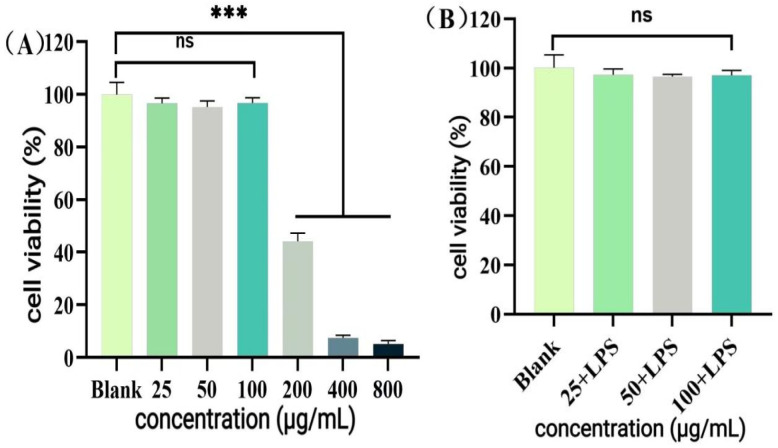
Effect of purified polyphenols from *C. oleifera* shell (P-CPCS) on the viability of RAW264.7 cells: (**A**) effect of P-CPCS on cell viability; (**B**) effect of P-CPCS on cell viability under oxidative stress. Note: *** *p* < 0.001, ns, not significant.

Cells were co-treated with 1 μg/mL LPS and P-CPCS to examine their effects on RAW264.7 cell viability. As shown in Figure 16A, 25–100 μg/mL P-CPCS had no significant effect on the cells within 24 h, and then cell viability decreased sharply. After 400 μg/mL, cell viability approached 0. Therefore, 25–100 μg/mL P-CPCS were chosen for the subsequent experiments. Figure 16B shows that co-treatment with 25–100 μg/mL P-CPCS and LPS for 24 h had no significant effect on cell viability, confirming the suitability of these conditions for further experiments.

**Figure 17:**
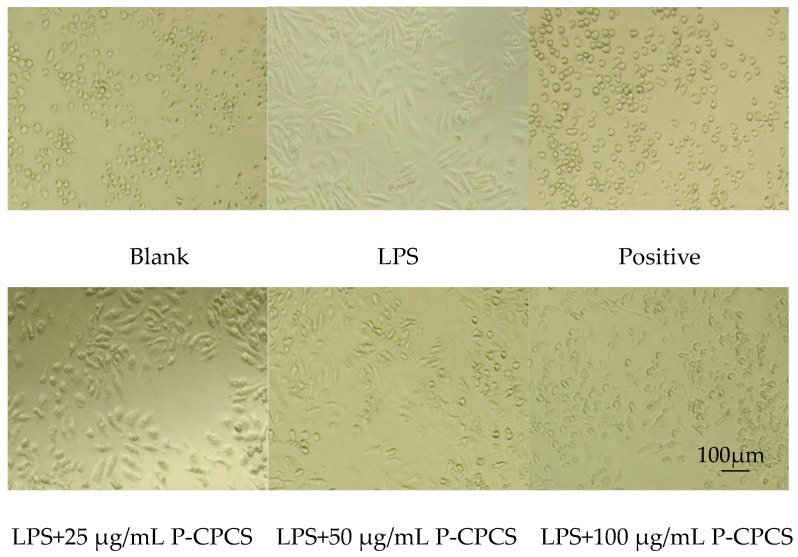
Effect of purified polyphenols from *C. oleifera* shell (P-CPCS) and LPS treatment on cell morphology (×40).

Under 40× inverted microscope observation (Figure 17), cells in the blank group were round/oval, with few pseudopods and good light transmission; the cells in the LPS group were obviously polarized, showing a spindle shape and reduced light transmission. The positive control group maintained normal cell morphology similar to the blank group, presenting predominantly round/oval shapes. Treatment with 25 μg/mL P-CPCS and 1 μg/mL LPS resulted in cell morphology comparable to the LPS-treated group without notable changes; after 50 μg/mL P-CPCS and 1 μg/mL LPS treatment, most cells were spindle-shaped, and a small number of round cells appeared, indicating partial inflammation alleviation; treatment with 100 μg/mL P-CPCS and 1 μg/mL LPS led to a significant reduction in spindle-shaped cells, accompanied by increased round cell numbers, demonstrating substantial mitigation of the inflammatory response.

**Figure 18:**
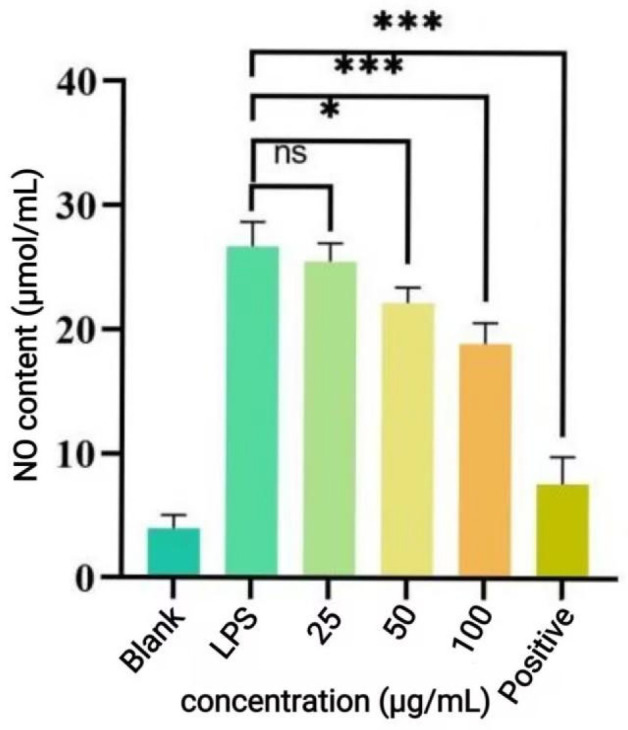
Effect of purified polyphenols from *C. oleifera* shell (P-CPCS) on the LPS-induced NO content in RAW264 7 cells. Note: *** *p* < 0.001, * *p* < 0.05; ns, not significant.

NO content in the reaction system was determined by Griess assay (Figure 18). LPS stimulation significantly increased NO levels compared to the blank group, indicating successful establishment of the inflammatory model, as cells exhibited inflammatory responses. 25 μg/mL P-CPCS treatment had no significant effect on NO production, demonstrating no alleviation of inflammation, which also coincided with the results presented in Figure 17. Treatment with 50 μg/mL and 100 μg/mL P-CPCS resulted in decreased NO content, suggesting that P-CPCS could partially inhibit NO production in RAW264.7 cells.

**Figure 19:**
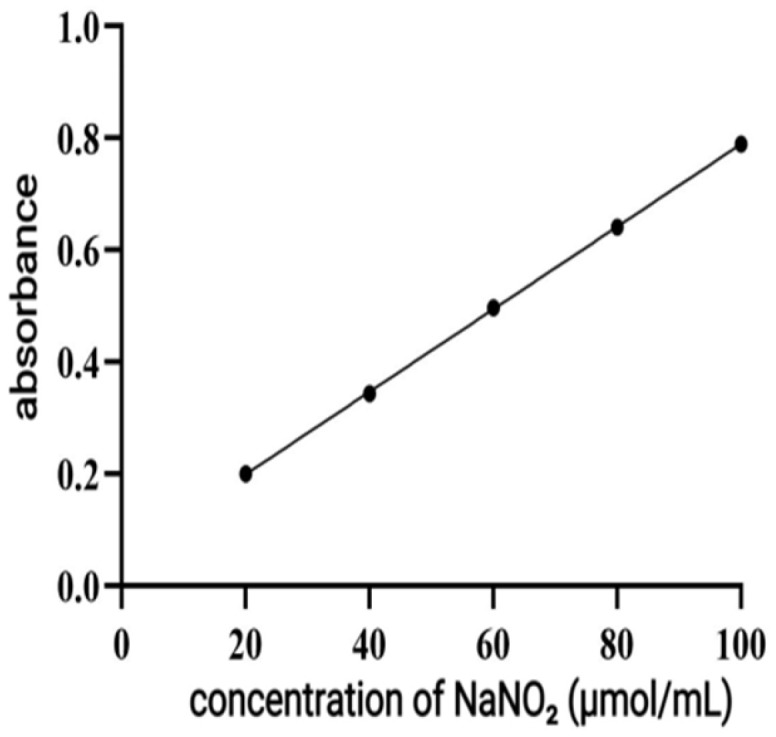
The NO standard curve.

According to the instructions of the assay kit, NaNO_2_ exhibited a strong linear relationship within the range of 20–100 μM. The fitted linear regression equation is y = 0.007378x + 0.05095, with R^2^ = 0.9999 (Figure 19). Therefore, P-CPCS can reduce the content of inflammatory factor NO in inflammation-damaged cells to a certain extent, restore RAW264.7 cells to normal morphology, and alleviate inflammatory injury.

#### 3.4.4. Tyrosinase Inhibition

**Figure 20:**
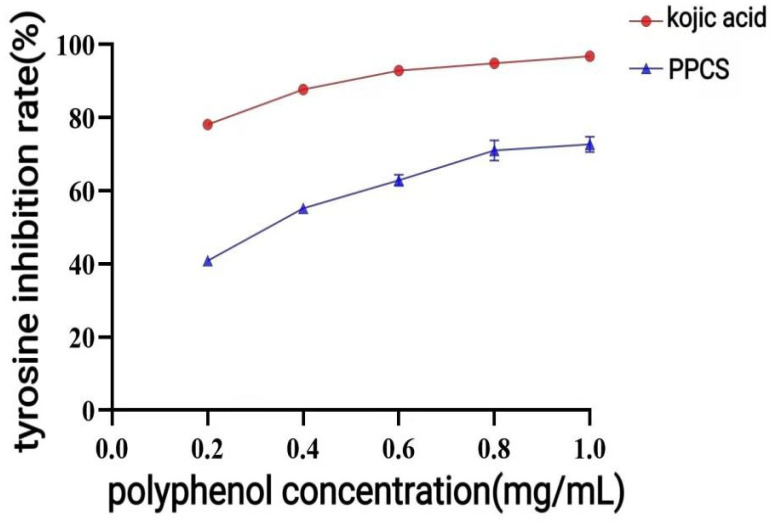
Rate of tyrosinase inhibition by purified polyphenols from *C. oleifera* shell (P-CPCS).

The enzymatic activity assay results (Figure 20) showed that the inhibition rate of the positive control group of kojic acid concentration no longer increased significantly after 0.6 mg/mL, and its IC_50_ was 0.07 mg/mL. P-CPCS had an inhibitory effect on tyrosinase, and there was a certain dose-effect relationship. The inhibition rate of the sample group no longer increased after 0.8 mg/mL, and it reached the maximum inhibition rate of 72.25%, and the IC_50_ was 0.31 mg/mL.

#### 3.4.5. Antibacterial Activity

**Figure 21:**
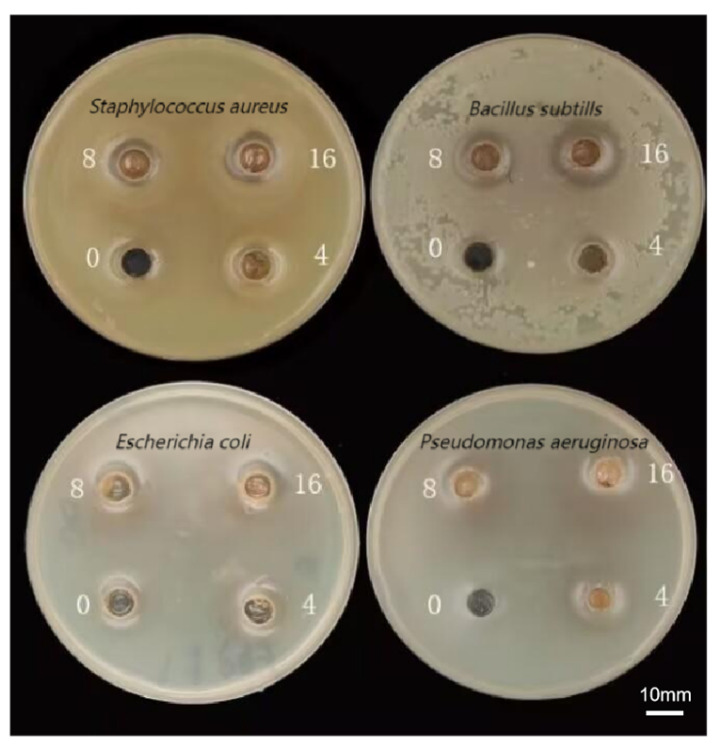
Diagram of purified polyphenols from *C. oleifera* shell (P-CPCS) on four bacterial species. The numbers 4, 8, and 16 indicate the concentrations of P-CPCS (in mg/mL), with 0 representing the blank control group (without P-CPCS).

P-CPCS exhibited broad-spectrum antibacterial effects against Gram-positive bacteria (Figure 21). As shown in Table 7, 4–16 mg/mL P-CPCS inhibited the growth of three Gram-positive bacteria: *Enterococcus*, *Bacillus subtilis*, and *Staphylococcus aureus*. The size of the inhibition zones increased with the increase in P-CPCS concentration. At a polyphenol concentration of 16 mg/mL, the maximum inhibition zones for *Enterococcus* and *Bacillus subtilis* reached 18.02 ± 1.02 mm. The positive control, rifampicin, and the sample solution did not inhibit the growth of three Gram-negative bacteria: *Escherichia coli*, *Salmonella*, and *Pseudomonas aeruginosa*. The MIC of P-CPCS against three bacterial strains is shown in Table 8 below: *Enterococcus* was inhibited at 0.25 mg/mL P-CPCS; *Bacillus subtilis* was inhibited at 2 mg/mL P-CPCS; and *Staphylococcus aureus* was inhibited at 0.5 mg/mL P-CPCS. The results demonstrate that P-CPCS exhibits relatively good antibacterial effects against these three Gram-negative bacteria. By comparing the MIC values, the inhibitory efficacy can be ranked as: *Staphylococcus aureus* > *Enterococcus* > *Bacillus subtilis*.

## 4. Discussion

Ultrasonic-assisted solvent extraction is an environmentally friendly method in which ultrasonic cavitation effectively disrupts the cell wall and enhances mass transfer, resulting in shorter processing time, lower solvent consumption, and higher extraction rate compared to traditional solvent extraction methods [19]. The correct selection of process parameters can maximize cost savings and improve the extraction efficiency. During the single-factor experiments for extraction optimization, the TPC values were observed to rise as the ultrasound power increased from 100 W to 270 W, beyond which further power increase resulted in a decline. This may be because increasing the ultrasonic power enhances the hydrodynamic effect, making it easier to rupture the cell wall and improve the yield [20]. However, excessive ultrasound power may lead to the formation of more bubbles in the solvent during cavitation, which may reduce the yield and effectiveness of ultrasound energy delivered to the medium [21]. Consistent with the influence of power, our preliminary experiments indicated that the yield of CPCS increased with rising ultrasonic temperature, peaking at 50 °C. This phenomenon may be attributed to enhanced solubility and dispersion of polyphenols in water at higher temperatures. However, high temperatures can easily lead to a decrease in the stability and biological activity of polyphenolic compounds and degradation [22]. The trend of increasing extraction yield within the ethanol concentration range of 0–40% can be attributed to the role of ethanol in disrupting hydrophobic interactions and hydrogen bonds between polyphenols and cellular structural components—such as polysaccharides and proteins—thereby improving the solubility and release of polyphenolic compounds [23]. However, when the ethanol concentration exceeded 40%, the extraction efficiency declined. This decrease may be due to the reduced polarity of the extraction solvent, which compromises its ability to dissolve polar polyphenols. Additionally, high ethanol levels could induce protein denaturation and promote the formation of insoluble complexes, further impeding mass transfer and solute diffusion [24]. The initial stage of ultrasonication time optimization revealed that the extraction yield increased with time due to enhanced cavitation and mechanical effects that promote cell wall rupture and solute diffusion [25]. Beyond 30 min, however, prolonged exposure likely induced oxidative degradation of thermolabile polyphenols [26]. In studying the influence of the L/M ratios, an appropriate increase in solvent volume was shown to improve mass transfer by reducing viscosity and enhancing interaction; however, excessively high ratios led to matrix over-swelling, potentially trapping compounds and lowering efficiency. Moreover, although ultrasound-assisted extraction (UAE) enhances the release of soluble components, an overly dilute system requires more energy and time to reach operational temperature, potentially leading to thermal degradation of polyphenols. Additionally, large solvent volumes can lead to saturated extraction solutions where further dissolution is limited, thereby lowering the overall yield per unit solvent [27]. Ding et al. compared the extraction efficiencies of different solvent systems for polyphenols from *C. oleifera* fruit hull [28]. They found that traditional ethanol extraction at 50% concentration yielded 28.4 mg/g. In contrast, a low eutectic solvent system reached 35.6 mg/g. In the present study, 38% ethanol was used to achieve an extraction rate of 40.05 ± 0.58 mg GAE/g dw. This result may be attributed to the precise optimization of the synergistic effect between ethanol concentration and the liquid-to-material ratio through response surface methodology. This finding is also consistent with the conclusion reported by Sun et al. [29], who stated that “an ethanol concentration of 30–40% provides the best polarity match for polyphenol extraction.” By optimizing the microwave-assisted ethanol extraction process for polyphenols from *C. oleifera* seed meal using the Box–Behnken model, they successfully increased the extraction yield from 26.5 mg/g to 38.9 mg/g. The higher extraction efficiency in our study may be due to the structural differences between materials. *C. oleifera* shells have a looser fiber structure than the seed meal. This structure makes it easier for ultrasonic cavitation to destroy the cell wall. It is also more conducive to polyphenol solubilization.

When macroporous resin was used for purification, the adsorption capacity increased with the rise in TPC at low sample concentrations, owing to the increased number of available active sites associated with polyphenols [30]. However, with further TPC increases, more impurities were adsorbed on the AB-8 resin, resulting in competition for active sites between the polyphenols and the impurities, which led to a slight drop in adsorption capacity. The pH of polyphenol solutions significantly influences both the molecular forms of polyphenols and their adsorption affinity to resins [31], thereby affecting the overall adsorption efficiency. As demonstrated in Figure 5, the adsorption rate decreased as pH increased, with the highest adsorption observed under acidic conditions at pH = 2. As demonstrated in Figure 5, the adsorption rate decreased as pH increased, with the highest adsorption observed under acidic conditions at pH = 2. This phenomenon can be attributed to the phenolic hydroxyl groups in the polyphenolic compounds, which tend to dissociate H^+^ ions and exhibit acidic properties. Under appropriately acidic conditions, the dissociation of H^+^ from polyphenols is suppressed, promoting their existence in neutral molecular (or salt) forms. This molecular state facilitates hydrogen bonding with the resin matrix, thereby enhancing the adsorption efficiency of macroporous resins. Conversely, in alkaline environments, OH^−^ ions neutralize the dissociated H^+^ from phenolic hydroxyl groups, converting polyphenols into anionic species. This ionic form weakens the physical interactions (e.g., hydrogen bonding and van der Waals forces) with the resin adsorption sites, ultimately reducing the adsorption capacity [31]. As shown in Figure 6, the yield started to drop as the ethanol concentration rose above 70%. This decline was caused by the growing polarity gap between polyphenols and solvents, which lowered polyphenol solubility [32]. In the early stage of the dynamic adsorption–desorption experiment, the macroporous resin exhibited complete adsorption of polyphenols due to its sufficient number of active sites. As the resin gradually reached saturation, its adsorption capacity diminished, resulting in an increase in leakage. A significant decrease in desorption rate (*p* < 0.05) was observed when the elution speed exceeded 1.5 mL/min, attributable to insufficient contact time for effective polyphenol elution. At the other extreme, an elution speed below 0.5 mL/min unnecessarily prolongs the process and increases the risk of polyphenol re-adsorption. After purification under optimized conditions using AB-8 macroporous resin, the polyphenol purity in CPCS increased substantially from 24.93% to 57.72%, with a recovery rate of 77.19%. These results confirm the significant purification effect of AB-8 resin on CPCS, which is likely attributable to the removal of competing components such as lipid-soluble impurities, thereby increasing the effective concentration of polyphenols.

The compositional analysis of P-CPCS was performed using HPLC-Q-Exactive-MS. Notably, the flavonoid profile comprised several subclasses with renowned bioactivities: Flavonol glycosides, including rutin, isoquercitrin, and hyperoside, are well-established potent antioxidants. Flavan-3-ols, such as proanthocyanidins [33], catechins [34], and epicatechins, are key antioxidants found abundantly in green tea. Aglycones: including quercetin [35], kaempferol, lignans, catechins [36], which are known for their broad anti-inflammatory and antimicrobial properties [37]. Furthermore, the identified phenolic acids, e.g., chlorogenic acid, neochlorogenic acid, caffeic acid, and gallic acid, are recognized for their ability to scavenge free radicals and enhance the body’s endogenous antioxidant defense systems [38]. The presence of this diverse spectrum of bioactive compounds, each with documented individual activities, strongly suggests that P-CPCS possesses a significant potential for exerting synergistic antioxidant, anti-inflammatory, and antimicrobial effects. Therefore, based on the identification of these active components and their known pharmacological mechanisms, this study conducted a systematic evaluation of the antioxidant, anti-inflammatory, antibacterial, and other biological activities of P-CPCS.

Consistent with the goal of enrichment, the purified P-CPCS exhibited markedly enhanced antioxidant capacity compared to the crude extract. The significantly lower IC_50_ values against ABTS^+^· and DPPH radicals demonstrate that the purification process successfully increased the specific activity. This boost in efficacy is likely due to the dual effect of concentrating known antioxidant polyphenols (e.g., chlorogenic acid) and removing inert impurities, confirming that the observed activity originates primarily from the polyphenolic components. These findings validate the purification strategy and highlight the potential of P-CPCS as a high-value, natural antioxidant for countering oxidative stress in various applications. The content of ROS in cells under oxidative stress was significantly increased, further damaging the cells. As tea polyphenols have been shown to mitigate H_2_O_2_-induced oxidative damage by enhancing SOD activity and reducing ROS [39], it is reasonable to hypothesize that the cytoprotective effect of P-CPCS observed in this study operates through a similar mechanism, potentially involving the activation of endogenous antioxidant enzymes and cellular defense systems. The precise molecular pathways, however, warrant further investigation. Macrophages are an important immune cell and play a pivotal role during inflammation in host defenses against pathogen infection. Lipopolysaccharide (LPS) can induce the polarization of RAW264.7 cells into M1-type cells, which are commonly used to construct cellular inflammation models. Inflammation, as the defensive response of body, is characterized by fluid accumulation and infiltration of inflammatory mediators (such as leukocytes) at the site of inflammation [40,41]. To evaluate the anti-inflammatory activity of P-CPCS, we employed a well-established cellular model wherein lipopolysaccharide (LPS) induces an inflammatory response in RAW264.7 macrophages. In this model, the overproduction of nitric oxide (NO) serves as a key indicator of inflammation. Our results demonstrate that P-CPCS significantly suppressed LPS-induced NO release, indicating potent anti-inflammatory efficacy. This anti-inflammatory activity is likely interrelated with the antioxidant properties of P-CPCS, given the pathological crosstalk between oxidative stress and inflammation. Excessive ROS generated during oxidative stress can activate pro-inflammatory signaling pathways like NF-κB [42,43], which in turn upregulates inducible NO synthase (iNOS) and further amplifies ROS production, creating a vicious cycle [44,45]. This study found that P-CPCS exhibit significant antioxidant and anti-inflammatory activity. Preliminary research suggests that these active components may exert their effects by regulating key intracellular signal transduction pathways. Studies have shown that flavonols such as quercetin and myricetin exhibit anti-inflammatory activity in RAW264.7 macrophages. Among these, quercetin may exert protective effects by activating the Nrf2 pathway and inhibiting the production of pro-inflammatory factors [46]; chlorogenic acid may regulate oxidative stress and inflammatory responses by modulating multiple signal pathways such as NF-κB and MAPK [47]; and proanthocyanidins may exert their effects by scavenging free radicals and regulating MAPK and other signaling pathways. These active components likely function via synergistic interactions affecting multiple targets and pathways, collectively constituting the mechanistic basis for the antioxidant and anti-inflammatory properties of CPCS. The potent inhibition of tyrosinase by P-CPCS suggests significant skin-whitening potential. Mechanistically, this activity may parallel that of oleuropein, which functions by downregulating key melanogenic proteins (TRP, TRP1, TRP2, and MITF) via modulation of the CREB and MAPK signaling pathways [48]. It is hypothesized that P-CPCS exhibits a strong skin-lightening activity via a mechanism analogous to that of oleuropein. P-CPCS exhibited notable antibacterial activity, particularly against Gram-positive bacteria. This effect can be mechanistically explained by its capacity to disrupt bacterial cell integrity. For instance, luteolin—a flavonoid present in P-CPCS—has been documented to cause significant damage to the cell wall and membrane in *Listeria* [49]. Consistent with this view, it is inferred that P-CPCS exerts its antibacterial action through a similar mechanism, likely involving dose-dependent disruption of membrane morphology, leading to cell lysis and leakage of intracellular contents. Gram-negative bacteria showed higher resistance, presumably owing to the Gram-negative outer membrane (OM) being impermeable to many molecules, and expression of numerous MDR efflux pumps that effectively reduce the intracellular concentration of the given drug [50].

## 5. Conclusions

This study developed an integrated cascade process combining ultrasound-assisted ethanol extraction—optimized using Box–Behnken design under fixed parameters (270 W ultrasound power, 38% ethanol concentration, 11 mL/g liquid-to-material ratio, 40 min extraction at 55 °C), with macroporous resin purification to significantly increase the yield of CPCS and obtain high-purity polyphenols. HPLC-Q-Exactive-MS analysis showed that the main active components of P-CPCS were flavonoids (rutin, quercetin) and organic acids (D-malic acid, citric acid). Research demonstrated that P-CPCS exhibits outstanding bioactivities, with particularly remarkable antioxidant properties (ABTS^+^·/DPPH radical scavenging, ·OH radical inhibition), bacteriostatic effects (against *Bacillus subtilis*, and *Staphylococcus aureus*, etc.), tyrosinase inhibition capacity and anti-inflammatory potential as evidenced by its suppression of LPS-induced NO overproduction in RAW264.7 macrophages. The novelty of this work lies in establishing an efficient and sustainable strategy for the valorization of *C. oleifera* shell, highlighting not only the enhancement of extraction efficiency and purity but also systematically elucidating the antioxidant mechanisms and performance of the purified polyphenols. The residual biomass after polyphenol extraction can be further utilized for lignin production, enabling comprehensive resource recovery and improving economic viability. Despite these promising results, challenges remain in evaluating the long-term antioxidant stability and interaction effects of P-CPCS in real application systems (emulsions, coatings, or biomaterials). Further studies are needed to fully clarify its in vivo antioxidant efficacy and biosafety through animal models, thereby facilitating its practical applications in functional foods, pharmaceuticals, and packaging materials.

## Figures and Tables

**Table 1:** Experimental design for single-factor optimization of polyphenol extraction.

Independent Variables	Levels				
	−2	−1	0	1	2
A: ultrasonic time (min)	10	20	30	40	50
B: ethanol volume fraction (%)	0	20	40	60	80
C: L/M ratio (g/mL)	5	10	15	20	25

**Table 2:** Independent variables and levels used in the BBD.

Independent Variables	Levels		
	−1	0	2
A: ultrasonic time (min)	20	30	40
B: ethanol volume fraction (%)	20	40	60
C: L/M ratio (g/mL)	5	10	15

**Table 3:** Tyrosinase inhibition test design.

System Components (μL)	0	1	2	3
	(Blank)	(Solvent Control)	(Sample)	(Background)
PBS buffer solution	100	150	/	50
L-dopa	50	50	50	50
tyrosinase	50	/	50	/
Sample/trehalose	/	/	100	100

**Table 4:** Box–Behnken Table design results.

Run No.	Factors			
	Time (min)	EtOH (%)	L/M Ratio (mL/g)	TPC (mg/g)
1	30	40	10	39.03
2	30	40	10	39.31
3	40	60	10	32.96
4	20	40	10	31.47
5	30	20	15	37.36
6	20	40	10	38.16
7	20	60	10	31.13
8	40	40	15	40.07
9	30	60	15	33.22
10	40	20	10	38.34
11	30	40	10	38.56
12	30	20	5	31.19
13	20	40	10	38.69
14	30	60	5	26.03
15	30	40	10	39.18
16	40	40	5	32.56
17	20	20	10	37.89

**Table 5:** Results of ANOVA for the response face regression model.

Source	Sum of Squares	df	Mean Square	F-Value	*p*-Value
Model	173.23	9	19.25	108.58	<0.0001 **
A-Time	7.35	1	7.35	41.46	0.0004 **
B-EtOH	3.78	1	3.78	21.31	<0.0024 **
C-SLR	33.84	1	33.84	190.89	<0.0001 **
AB	0.091	1	0.091	0.51	0.4971 ns
AC	1.24	1	1.24	7.02	0.0330 **
BC	0.19	1	0.19	1.10	0.3297 ns
A2	1.43	1	1.43	8.09	0.0249 *
B2	56.11	1	56.11	316.51	<0.0001 **
C2	59.98	1	59.98	338.38	<0.0001 **
Residual	1.24	7	0.18		
Lost Proposal	0.69	3	0.23	1.67	0.3096 ns
Pure Error	0.55	4	0.14		
Aggregate	174.47	16			

Note: ** extremely significant (*p* < 0.01); * significant (*p* < 0.01); ns, no significant (*p* > 0.05).

**Table 6:** Results of chemical composition identification of polyphenols from purified polyphenols from *C. oleifera* shell (P-CPCS).

No.	Compound	Formula	Ion Mode	RT (min)	Theoretical Value	Measured Value	PPM
1	rutin	C_27_H_30_O_16_	M+H	4.82	609.14612	609.1473	−1.93714
2	gallic acid	C_21_H_20_O_11_	M+H	2.69	169.01425	169.0134	5.02916
3	proanthocyanidin	C_30_H_26_O_13_	M+H	3.19	595.14459	595.1481	−5.89773
4	hyperoside	C_21_H_20_O_11_	M-H	5.06	447.094	447.09329	1.58803
5	catechin	C_15_H_14_O_6_	M+H	3.87	291.0862	291.086	0.68708
6	licorice flavanone	C_20_H_20_O_5_	M+H	6.04	341.14001	341.139	2.96066
7	isoquercitrin	C_21_H_20_O_12_	M-H	4.86	463.0874	463.0882	−1.72754
8	phloridzin	C_21_H_24_O_10_	M-H	4.95	435.12967	435.1295	0.39069
9	neochlorogenic acid	C_16_H_18_O_9_	M+H	3.49	355.10001	355.101	−2.78795
10	kaempferol	C_15_H_10_O_6_	M+H	5.67	287.05502	287.0555	−1.67215
11	quercetin	C_15_H_10_O_7_	M+H	6.93	303.04993	303.0505	−1.88088
12	chlorogenic acid	C_16_H_18_O_9_	M+H	4.07	353.08621	353.0878	−4.50315
13	ferulic acid	C_10_H_10_O_4_	M-H	4.48	193.05063	193.05009	2.79719
14	quercitrin	C_21_H_20_O_11_	M+H	5.06	449.10782	449.1083	−1.06879
15	epicatechin	C_15_H_14_O_6_	M+H	4.51	291.13519	291.1345	2.30449
16	licoflavone C	C_20_H_18_O_5_	M+H	4.59	361.10501	361.1036	3.90468
17	avicularin	C_20_H_18_O_11_	M-H	4.97	433.07764	433.0788	−2.67850
18	skimmin	C_15_H_16_O_8_	M+H	3.13	325.09	325.0902	−0.61521
19	luteolin	C_15_H_10_O_6_	M+H	4.70	287.05502	287.0556	−2.02052
20	caffeic acid	C_10_H_10_O_4_	M+H	6.67	181.04953	181.0497	−0.93897

**Table 7:** The antibacterial activity of purified polyphenols from *C. oleifera* shell (P-CPCS) against six bacterial species.

Strains	Inhibition Zone					
	Ampicillin Sodium	Rifampicin	4 mg/mL	8 mg/mL	16 mg/mL	Blank Control
*Bacillus subtilis*	21.45 ± 0.9	21.16 ± 1.51	10.58 ± 1.06	14.51 ± 1.20	15.72 ± 1.02	8.00 ± 0.00
*Pseudomonas aeruginosa*	13.89 ± 0.39	8.00 ± 0.00	8.00 ± 0.00	8.00 ± 0.00	8.00 ± 0.00	
*Staphylococcus aureus*	35.06 ± 3.46	23.63 ± 0.69	13.06 ± 0.69	14.51 ± 0.73	16.00 ± 0.92	
*Escherichia coli*	18.31 ± 1.40	8.00 ± 0.00	8.00 ± 0.00	8.00 ± 0.00	8.00 ± 0.00	

**Table 8:** Minimum inhibitory concentration (MIC values) of bacteria.

Strains	CPCS Concentration (mg/mL)							
	0.125	0.25	0.5	1	2	4	8	16
*Bacillus subtilis*	+	+	+	+	-	-	-	-
*Staphylococcus aureus*	+	+	-	-	-	-	-	-

Note: “+” means the liquid is cloudy; “-“ means the liquid is clear.

## Data Availability

The data presented in this study are available on request from the corresponding author. The data are not publicly available due to privacy and ethical restrictions.
